# Cardiovascular magnetic resonance to differentiate veteran athlete’s heart with cavity dilatation and mild dilated cardiomyopathy

**DOI:** 10.1093/ehjci/jeaf234

**Published:** 2025-08-09

**Authors:** Wasim Javed, Raluca Tomoaia, Maryum Farooq, Bradley Chambers, Ioannis Botis, Ze M Goh, Benjamin Brown, Louise A E Brown, Jonathan Farley, Hui Xue, Eylem Levelt, Erica Dall’Armellina, John P Greenwood, Peter Kellman, Sven Plein, Peter P Swoboda

**Affiliations:** Department of Biomedical Imaging Science, Leeds Institute of Cardiovascular and Metabolic Medicine, University of Leeds, Leeds LS2 9JT, UK; Cardiology Department, Iuliu Hațieganu, University of Medicine and Pharmacy, Cluj-Napoca, Romania; Department of Biomedical Imaging Science, Leeds Institute of Cardiovascular and Metabolic Medicine, University of Leeds, Leeds LS2 9JT, UK; Department of Biomedical Imaging Science, Leeds Institute of Cardiovascular and Metabolic Medicine, University of Leeds, Leeds LS2 9JT, UK; Department of Biomedical Imaging Science, Leeds Institute of Cardiovascular and Metabolic Medicine, University of Leeds, Leeds LS2 9JT, UK; Department of Biomedical Imaging Science, Leeds Institute of Cardiovascular and Metabolic Medicine, University of Leeds, Leeds LS2 9JT, UK; Department of Biomedical Imaging Science, Leeds Institute of Cardiovascular and Metabolic Medicine, University of Leeds, Leeds LS2 9JT, UK; Department of Biomedical Imaging Science, Leeds Institute of Cardiovascular and Metabolic Medicine, University of Leeds, Leeds LS2 9JT, UK; Department of Biomedical Imaging Science, Leeds Institute of Cardiovascular and Metabolic Medicine, University of Leeds, Leeds LS2 9JT, UK; Cardiovascular Branch, National Institutes for Health, National Heart, Lung, and Blood Institute, Bethesda, USA; Cardiometabolic Imaging Department, Baker Heart and Diabetes Institute & University of Melbourne, Melbourne, Australia; Department of Biomedical Imaging Science, Leeds Institute of Cardiovascular and Metabolic Medicine, University of Leeds, Leeds LS2 9JT, UK; Cardiometabolic Imaging Department, Baker Heart and Diabetes Institute & University of Melbourne, Melbourne, Australia; Cardiovascular Branch, National Institutes for Health, National Heart, Lung, and Blood Institute, Bethesda, USA; Department of Biomedical Imaging Science, Leeds Institute of Cardiovascular and Metabolic Medicine, University of Leeds, Leeds LS2 9JT, UK; Department of Biomedical Imaging Science, Leeds Institute of Cardiovascular and Metabolic Medicine, University of Leeds, Leeds LS2 9JT, UK

**Keywords:** myocardial fibrosis, athlete’s heart, dilated cardiomyopathy, parametric tissue characterization

## Abstract

**Aims:**

This study aimed to investigate the distribution of myocardial fibrosis and patterns of tissue characteristics on cardiovascular magnetic resonance (CMR) between athletes with left ventricular (LV) dilatation and mild dilated cardiomyopathy (DCM) patients.

**Methods and results:**

We prospectively recruited male cyclists/triathletes aged ≥50 years who undertook ≥10 h/week of exercise for ≥15 years along with age-/sex-matched patients with non-ischaemic heart failure (HF). Participants underwent clinical assessment, 12-lead ECG, stress-perfusion CMR with fibrosis assessment, and parametric tissue mapping.

Following CMR, included participants in both groups had left ventricular ejection fraction (LVEF) > 40% and left ventricular end-diastolic volume indexed to body surface area (LVEDVi) ≥ 110 mL/m^2^ without ischaemic heart disease or significant cardiac pathology on CMR likely to cause HF. Of 113 participants (64 athletes and 49 mild DCM patients), athletes with fibrosis demonstrated a greater prevalence of inferolateral fibrosis (87.5% vs. 50.0%, *P* = 0.002), whereas inferoseptal fibrosis was more common in mild DCM patients (45.8% vs. 9.4%, *P* = 0.002). Native T1 (1249.0 ± 38.1 vs. 1308.3 ± 47.1 ms, *P* < 0.001) and extracellular volume (ECV) (22.0 ± 2.1 vs. 25.9 ± 3.5%, *P* < 0.001) were lower in athletes. Athletes had greater right ventricular end-diastolic volume indexed to body surface area (RVEDVi) (121.0 ± 14.3 vs. 97.6 ± 25.2%, *P* < 0.001), myocardial perfusion reserve (MPR) (3.65 ± 1.30 vs. 2.76 ± 0.92, *P* < 0.001), and stress myocardial blood flow (MBF) (2.09 ± 0.70 vs. 1.62 ± 0.66, *P* < 0.001) than mild DCM patients. On receiver-operator curve analysis, native T1 [area under the curve (AUC) 0.89, *P* < 0.001], ECV (AUC 0.85, *P* < 0.001), RVEDVi (AUC 0.81, *P* < 0.001), and stress MBF (AUC 0.68, *P* = 0.002) were able to differentiate between groups.

**Conclusion:**

Septal fibrosis is rare amongst veteran athletes with LV dilation in contrast to mild DCM patients. Native T1, ECV, and RVEDVi can also discriminate between these overlapping phenotypes, which may be clinically useful.

## Graphical Abstract

**Graphical Abstract:**
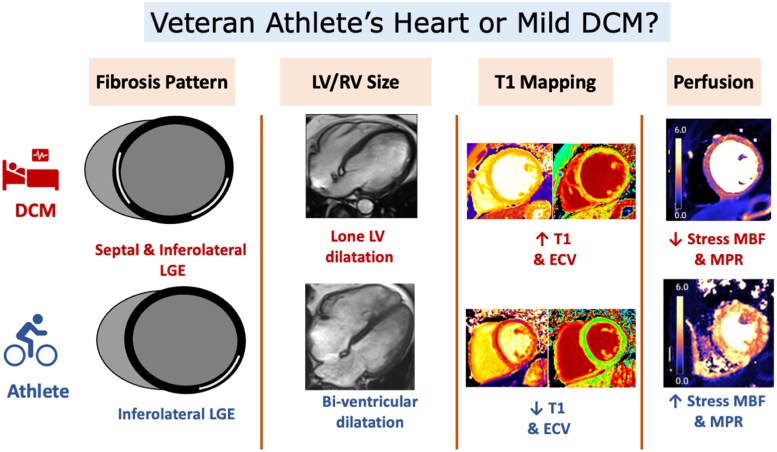
Cardiovascular magnetic resonance (CMR) characteristics of veteran athlete’s heart (bottom panel) with left ventricular (LV) dilatation vs. mild dilated cardiomyopathy (DCM) (top panel). Left hand column, schematic fibrosis patterns; left centre column, four-chamber view; right centre column, native T1 and ECV maps; right hand column, quantitative perfusion maps. ECV, extracellular volume; LGE, late gadolinium enhancement; LV, left ventricular; MBF, myocardial blood flow; MPR, myocardial perfusion reserve.

## Introduction

Endurance athletes often undergo cardiovascular adaptions that lead to an ‘athlete’s heart’ phenotype, which includes left ventricular (LV) dilatation and mildly reduced left ventricular ejection fraction (LVEF) at rest. In elite athletes, the degree of LV dilatation may be marked as demonstrated by a study involving 286 former Tour De France cyclists, where half of the cohort exhibited LV diastolic diameters >60 mm on echocardiography.^1^ Furthermore, one-third of the cyclists with significant LV enlargement concurrently had reduced LVEF at rest. Such LV adaptation is thought to occur due to the exponential increase in LV stroke volume during high-intensity aerobic exercise. Male endurance athletes who simultaneously undertake a high degree of isometric exercise such as cyclists, rowers, and swimmers are believed to experience the greatest extent of LV remodelling.^2,3^

Dilated cardiomyopathy (DCM) is also characterized by LV enlargement in combination with reduced LVEF.^4^ Differentiation of athlete’s heart from mild forms of DCM can be challenging, in particular in older athletes with cardiac risk factors. Furthermore, isolated LV dilatation is recognized as an important preclinical phase of certain DCM phenotypes.^5^ In clinical practice, response to exercise may be used to differentiate athlete’s heart from DCM. Healthy athletes who demonstrate physiological mildly depressed LV systolic function at rest exhibit an increase in LV function during exercise, and a period of detraining can reduce LV cavity size amongst certain athletes.^6,7^ However, DCM patients may also significantly improve their VO_2_max during exercise.^8^ Additionally, LVEF may improve up to 20% during acute physical exertion in DCM patients, which further blurs the distinction between those with mild DCM and athlete’s heart when using exercise testing to differentiate these groups.^9^ Furthermore, athletes may be hesitant to de-train, and access to exercise assessment facilities is not ubiquitous, calling for other methods to reliably distinguish between mild DCM and athlete’s heart. It is important to distinguish these two entities to avoid athletes being erroneously labelled with a pathological cardiac disorder with sporting implications whilst also enabling those with mild DCM to be correctly identified and receive early treatment.

In DCM, the presence of septal non-ischaemic myocardial fibrosis on cardiovascular magnetic resonance (CMR) has been shown to be independently associated with adverse prognosis, which is potentiated when combined with myocardial fibrosis of the LV lateral wall.^10,11^ Myocardial fibrosis has also been increasingly detected in lifelong endurance athletes, particularly those who are older males, the significance of which is debated.^12^ The specific patterns of myocardial fibrosis and parametric tissue characteristics between those with mild DCM and veteran endurance athletes with cavity dilatation have not been directly compared to determine if they can improve the differentiation of these two populations.

We aimed to compare the myocardial fibrosis distribution and tissue characteristics between athletic LV dilatation and mild DCM using advanced CMR-late gadolinium enhancement (LGE) imaging and parametric tissue characterization techniques.

## Methods

This study was undertaken in accordance with the Declaration of Helsinki and was granted ethical approval by the South Yorkshire & Humber NHS Research Ethics Committee and Health Research Authority (21/YH/0231 and 17/YH/0300). Each participant provided written informed consent prior to taking part in the research. All study investigations and participant visits were undertaken at the University of Leeds Advanced Imaging Centre, Leeds General Infirmary, Leeds, UK.

### Recruitment

One hundred eighty-one male endurance athletes who were aged ≥50 years old were prospectively recruited from sporting clubs/organizations within the UK via email invitation to their respective club/organization. Athletes undertook ≥10 h per week of formal exercise for ≥15 years and competed regularly either at the local, national, or international level.

Patients with mild DCM were selected from a larger cohort of 733 patients with a clinical diagnosis of heart failure (HF) and impaired LV function (LVEF < 50%) on echocardiography, recruited in the preceding 12 months. From this cohort, 375 males were included in this study to match the sex of the athletes.

From these two cohorts, participants with LVEF ≥ 40% and left ventricular end-diastolic volume indexed to body surface area (LVEDVi) ≥ 110 mL/m^2^ were selected for this study based on European Society of Cardiology guidelines defining a mildly reduced EF as LVEF 41–49% and the CMR reference for LV dilatation.^13,14^ Exclusion criteria for both cohorts included known ischaemic heart disease (IHD), symptoms of chest pain, severe valvular heart disease, myocarditis, and hypertrophic cardiomyopathy. Participants were also excluded if they had cardiac amyloid, inducible ischaemia, or myocardial infarction (MI) on CMR.

### Study procedures

All participants underwent baseline assessment consisting of physical examination along with documentation of their medical and lifestyle history.

Physical examination involved measurement of height, weight, resting blood pressure (BP), and heart rate (HR). Blood sampling for full blood count, renal profile, lipid profile, and glycated haemoglobin was undertaken for haematocrit measurement and to identify the presence of hyperlipidaemia, diabetes mellitus, or kidney disease. All participants underwent resting 12-lead ECG (MAC500, GE Medical Systems, Milwaukee, WI, USA) to identify pre-existing cardiac disease and rhythm abnormalities.

### CMR protocol

All participants underwent CMR imaging with an identical protocol (Siemens Prisma 3.0 T CMR scanner, Siemens Healthineers, Erlangen, Germany). Participants were advised to avoid caffeine for 24 h before the study. The CMR scan protocol consisted of the following:

- Cine imaging in short axis (SAX) and multiple long axis (LAX) planes for volumetric analysis
- Adenosine stress and rest quantitative myocardial perfusion to identify myocardial ischaemia and microvascular function
- Pre- and post-contrast T1 mapping to allow estimation of the myocardial extracellular volume (ECV) fraction
- T2 mapping to identify inflammation and oedema
- Motion-corrected (MOCO) bright and dark-blood LGE in SAX and multiple LAX planes to identify and quantify LV fibrosis

For perfusion imaging, a free-breathing MOCO dual sequence single bolus perfusion sequence was used to provide pixel wise mapping of myocardial blood flow (MBF).^15^ A 140 µg/kg/min of adenosine was administered through a peripheral intravenous cannula for 3 min or increased to 210 µg/kg/min if there was a lack of haemodynamic response. BP was recorded every 2 min, and continuous ECG monitoring was utilized throughout. When the HR increased by >10% compared with baseline accompanied with symptoms of adenosine-induced hyperaemia, a bolus of 0.05 mmol/kg non-ionic gadolinium-based contrast (Gadovist®, Bayer, Leverkusen, Germany) was given followed by a 20 mL saline flush using an automated injection pump (Medrad MRXperion Injection System, Bayer, Leverkusen, Germany) for both stress and rest imaging.^16^ Perfusion maps were acquired at three LV SAX 8 mm slices at the basal, mid, and apical levels with slice spacing varying on a per-patient basis to cover the LV.

Pre-contrast native T1 maps were acquired in three 8 mm LV SAX slices (basal, mid, and apical) with planning identical to perfusion slices using a breath-held 5s3s Modified Look-Locker inversion recovery (MOLLI) acquisition. T2 maps were acquired at the exact same LV SAX slice locations using a breath-held T2-prepared spoiled gradient echo (GE) pulse sequence resulting in single-shot T2-prepared images.

LGE images were acquired in a SAX stack covering the entire LV along with four-, three-, and two-chamber views using a free-breathing MOCO T1-weighted, inversion-recovery sequence. A top-up of 0.1 mmol/kg gadolinium-based contrast agent (Gadovist®, Bayer, Leverkusen, Germany) was given immediately following rest perfusion imaging. Post-contrast T1 mapping was performed exactly 15 min after contrast administration using 4s(3s)3s(3s)2s MOLLI with identical positioning and planning to the native T1 mapping. A single slice of dark-blood LGE was performed at the SAX mid-LV to image both papillary muscles.

### CMR analysis

All CMR studies were analysed using commercially available software (cvi42, Circle Cardiovascular Imaging Inc. Calgary, Canada). Volumetric data were calculated by tracing the LV (endocardial and epicardial borders excluding papillary muscles), right ventricle (RV) (endocardial borders), and left atrium (LA). LV/RV volumes, LV mass, and LA volumes were indexed to body surface area (BSA). LA volumes were produced by semi-automated tracing of the LA endocardial border in the four- and two-chamber cine views using the biplane area-length method. LV global longitudinal strain (GLS) was performed by manually tracing the LV endocardial and epicardial borders in end-diastole using the four-, three-, and two-chamber cine LAX images before using semi-automated feature tracking software to track the contours throughout the cardiac cycle.

Visual assessment of regional ischaemia in a coronary distribution was performed from stress and rest perfusion images. Automated quantitative assessment of MBF at stress and rest was performed using a previously validated method.^17^ This provided automated calculation of global and segmental MBF, myocardial perfusion reserve (MPR), and endo-epicardial MBF gradient.

T1 and T2 maps were analysed by manually contouring the LV mid-slice endocardial and epicardial borders with a 15% offset applied. T1 measurements were performed on the same mid-LV slice for native T1 pre- and post-contrast along with corresponding blood pool. ECV was calculated using the formula:


$$ECV=\;(1-haematocrit)\;\frac{\left(\Delta R1myocardium\right)}{\left(\Delta R1blood\right)}\;$$

The presence of focal LV fibrosis was confirmed only when an area of LGE was visualized on an LV SAX stack image along with corresponding orthogonal LV LAX plane and/or matching ECV map images. Segmental quantification was performed on LGE LV SAX stack images by contouring LV endocardial and epicardial borders on those slices containing LGE using the five-standard deviation method to provide numerical quantitative LV fibrosis in grams. The five-standard deviation method was chosen to avoid overestimating the presence of fibrosis, particularly where fibrosis was subtle.^18^ Segmental quantitative fibrosis assessment was performed by sub-dividing each myocardial segment of the conventional 16 myocardial segment model into three further segments; subepicardial, mid-myocardial, and subendocardial, leading to 48 myocardial sub-segments. The LV fibrosis percentage of the total myocardium was calculated by dividing the total LV fibrosis by the LV mass. RV insertion point (RVIP) LGE was noted but not classified as fibrosis. The presence of papillary muscle fibrosis was confirmed using a single slice of dark-blood LGE at the mid-LV level.

### Statistical analysis

Statistical analyses for all studies were undertaken using SPSS statistics 29 (IBM SPSS, Armonk, New York, USA). Normality of data was assessed using the Shapiro–Wilk test. Continuous data were presented as mean ± standard deviation or median ± interquartile range depending on the normality of the data. Categorical data were presented as frequency (percentage). Continuous variables were compared using the unpaired *t*-test or Mann–Whitney *U* test depending on the normality of data. Categorical variables were compared using χ^2^ test. Depending upon normality of data, either Pearson’s or Spearman’s correlation coefficient was used to assess correlation. C-statistics were used to perform receiver operating characteristic (ROC) curve analysis. A *P* value of <0.05 was considered statistically significant in all analyses.

## Results

**Figure 1:**
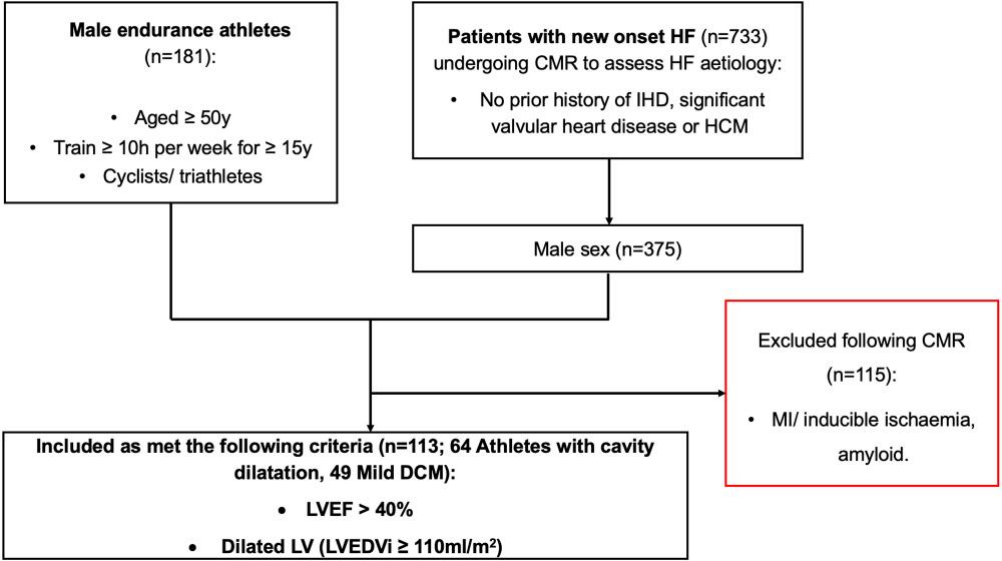
Consolidated Standards Of Reporting Trials diagram. Flow chart of participant recruitment for male veteran athletes with cavity dilatation and patients with mild DCM. CMR, cardiac magnetic resonance; DCM, dilated cardiomyopathy; IHD, ischaemic heart disease; HCM, hypertrophic cardiomyopathy; HF, heart failure; IHD, ischaemic heart disease; LVEDVi, left ventricular end-diastolic volume indexed to body surface area; LVEF, left ventricular ejection fraction; MI, myocardial infarction; y, years.

The final analysis included 113 participants (64 athletes and 49 mild DCM patients) after identifying male athletes and HF patients with an LVEDVi ≥ 110 mL/m^2^ and an LVEF ≥ 40% on CMR (*Figure 1*).^19^

Athletes had lower body mass index (BMI), HR, and BP along with a lower incidence of pre-existing hypertension, hyperlipidaemia, and stroke than patients with mild DCM (*Table 1*).

**Table 1:** Baseline assessment and demographic data according to athletes vs. mild DCM patients

	Athletes (*n* = 64)	Mild DCM (*n* = 49)	*P* value
Age (years)	58.8 ± 6.0	56.8 ± 13.4	0.31
BMI (kg/m^2^)	24.4 ± 2.2	30.5 ± 16.5	**0**.004*
Resting HR (BPM)	51.6 ± 8.0	62.5 ± 13.0	**<0**.001*
Systolic BP (mmHg)	117.7 ± 18.8	131.1 ± 23.0	**0**.001*
Diastolic BP (mmHg)	72.7 ± 8.2	76.9 ± 11.1	**0**.03*
Diabetes (*n*)	0	3 (6.1%)	0.05
Hypertension (*n*)	1 (1.6%)	21 (42.9%)	**<0**.001*
Stroke/TIA (*n*)	0	6 (12.2%)	**0**.004*
AF (*n*)	8 (12.5%)	10 (20.4%)	0.24
Hyperlipidaemia (*n*)	2 (3.1%)	10 (20.4%)	**0**.003*
Current smoker (*n*)	1 (1.6%)	8 (16.3%)	**0**.02*
Ex-smoker (*n*)	6 (9.4%)	14 (28.6%)	0.05
NYHA I	N/A	34 (69.4%)	
Antiplatelet (*n*)	0	7	0.002*
Beta-blocker (*n*)	2	37	<0.001*
ACE-i/ARB (*n*)	0	38	<0.001*
MRA (*n*)	0	13	<0.001*
Diuretic (*n*)	0	15	<0.001*
Statin (*n*)	3	21	<0.001*
Oral anticoagulant (*n*)	3	9	0.02*
SGLT2 inhibitor (*n*)	0	7	0.002*
Oral hypoglycaemic (*n*)	0	7	0.002*

Values are mean ± standard deviation or frequency (%). * Bold values denote *P* < 0.05.

AF, atrial fibrillation; BMI, body mass index; BP, blood pressure; BPM, beats per minute; DCM, dilated cardiomyopathy; NYHA, New York Heart Association; TIA, transient ischaemic attack.

LVEDVi (123.3 ± 12.6 mL/m^2^ vs. 129.8 ± 23.1 mL/m^2^, *P* = 0.06) and LV mass indexed to BSA (LVMi) (78.0 ± 10.6 g/m^2^ vs. 78.9 ± 17.9 g/m^2^, *P* = 0.73) were not significantly different between athletes and mild DCM patients. However, LVEF (52.0 ± 6.1% vs. 47.6 ± 5.2%, *P* < 0.001) and right ventricular end-diastolic volume indexed to BSA (RVEDVi) (121.0 ± 14.3 mL/m^2^ vs. 97.6 ± 25.2 mL/m^2^, *P* < 0.001) were both greater in athletes than mild DCM patients (*Table 2*).

**Table 2:** CMR volumetric parameters according to athletes vs. mild DCM patients

	Athletes (*n* = 64)	Mild DCM (*n* = 49)	*P* value
LVEDV (ml)	241.7 ± 32.4	270.2 ± 49.6	**<0**.001*
LVEDVi (mL/m^2^)	123.3 ± 12.6	129.8 ± 23.1	0.06
LVEF (%)	55.4 ± 4.3	47.6 ± 5.2	**<0**.001*
LVM (g)	152.7 ± 22.5	164.3 ± 36.6	**0**.04*
LVMi (g/m^2^)	78.0 ± 10.6	78.9 ± 17.9	0.73
LVM/LVEDV	0.63 ± 0.07	0.61 ± 0.12	0.13
LV GLS (%)	−14.9 ± 2.4	−11.8 ± 3.4	**<0**.001*
RVEDV (ml)	237.1 ± 33.1	202.9 ± 53.1	**<0**.001*
RVEDVi (mL/m^2^)	121.0 ± 14.3	97.6 ± 25.2	**<0**.001*
RVEDV/LVEDV	0.98 ± 0.09	0.75 ± 0.16	**<0**.001*
RVEF (%)	52.4 ± 5.3	54.7 ± 10.3	0.12
Interventricular septum MWT (mm)	9.7 ± 1.4	10.0 ± 2.2	0.32
Basal inferolateral MWT (mm)	7.2 ± 1.3	6.7 ± 1.9	0.10
LAV (mL)	110.4 ± 31.6	102.5 ± 46.8	0.29
LAVi (mL/m^2^)	56.4 ± 15.6	53.2 ± 25.5	0.41

Values are mean ± standard deviation. * Bold values denote *P* < 0.05.

LAV, left atrial volume; LAVi, left atrial volume indexed to body surface area; LVEDV, left ventricular end-diastolic volume; LVEDVi, left ventricular end-diastolic volume indexed to body surface area; LVEF, left ventricular ejection fraction; LVM, left ventricular mass; LVMi, left ventricular mass indexed to body surface area; MWT, maximum wall thickness; RVEDV, right ventricular end-diastolic volume; RVEDVi, right ventricular end-diastolic volume indexed; RVEF, right ventricular ejection fraction.

LV GLS values were more significantly negative in athletes with cavity dilatation compared with mild DCM patients (−14.9 ± 2.4% vs. −11.8 ± 3.4%, *P* < 0.001). However, both groups had reduced longitudinal function compared with normal reference ranges.^19^ There were no differences in LA volume indexed to BSA (LAVi) between athletes and mild DCM patients (56.4 ± 15.6 mL/m^2^ vs. 53.2 ± 25.5 mL/m^2^, *P* = 0.41).

**Figure 2:**
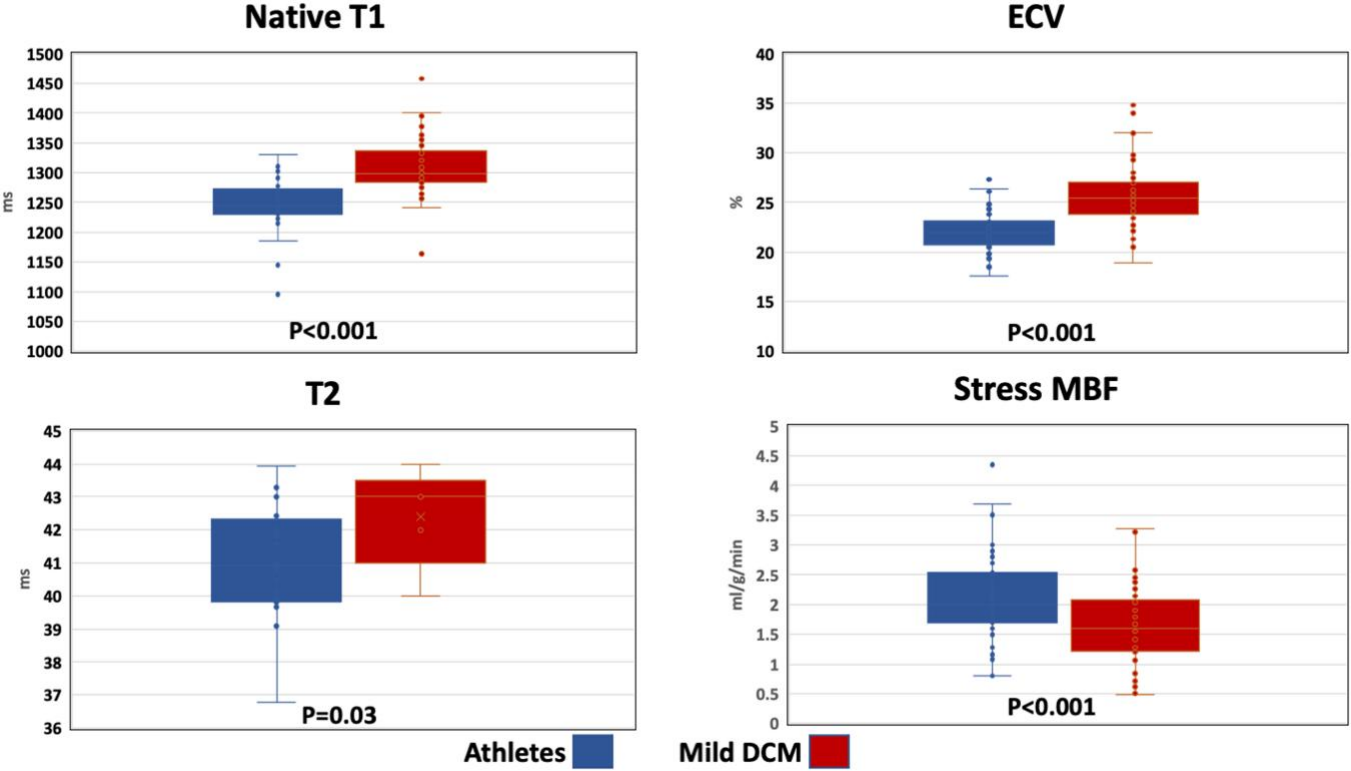
Box plots demonstrating differences in parametric tissue mapping between athletes (left) and mild DCM patients (right). Athletes had lower ECV, native T1, and T2 but higher stress MBF than patients with mild DCM. ECV, extracellular volume; DCM, dilated cardiomyopathy; MBF, myocardial blood flow.

Native T1 (1249.0 ± 38.1 ms vs. 1308.3 ± 47.1 ms, *P* < 0.001) and ECV (22.0 ± 2.1% vs. 25.9 ± 3.5%, *P* < 0.001) were both lower in athletes than mild DCM patients (*Table 3*). Furthermore, athletes had higher MPR (3.65 ± 1.30 vs. 2.76 ± 0.92, *P* < 0.001) and stress MBF (2.09 ± 0.70 mL/g/min vs. 1.62 ± 0.66 mL/g/min, *P* < 0.001) than mild DCM patients, but there was no significant difference in resting MBF (0.61 ± 0.27 mL/g/min vs. 0.61 ± 0.17 mL/g/min, *P* = 0.93) between the groups (*Figure 2*).

**Table 3:** CMR fibrosis, parametric tissue mapping, and quantitative perfusion data according to athletes vs. mild DCM patients

	Athletes (*n* = 64)	Mild DCM (*n* = 49)	*P* value
Stress MBF (mL/g/min)	2.09 ± 0.70	1.62 ± 0.66	**<0**.001*
Rest MBF (mL/g/min)	0.61 ± 0.27	0.61 ± 0.17	0.93
MPR	3.65 ± 1.30	2.76 ± 0.92	**<0**.001*
Native T1 (ms)	1249.0 ± 38.1	1308.3 ± 47.1	**<0**.001*
ECV (%)	22.0 ± 2.1	25.9 ± 3.5	**<0**.001*
T2 (ms)	40.8 ± 2.0	41.8 ± 3.0	**0**.03*
Non-ischaemic fibrosis (*n*)	32 (50%)	24 (49.0%)	0.92
Fibrosis mass (g)	3.5 ± 2.9	7.4 ± 12.0	0.09
Fibrosis mass/LV mass (%)	3.5 ± 2.9	7.4 ± 12.0	0.09
RVIP LGE (*n*)	43 (67.2%)	3 (6.1%)	**<0**.001*
Papillary fibrosis (*n*)	20 (31.3%)	11 (22.4%)	0.30

Values are mean ± standard deviation. * Bold values denote *P* < 0.05.

ECV, extracellular volume; LGE, late gadolinium enhancement; LV, left ventricular; MBF, myocardial blood flow; MPR, myocardial perfusion reserve.

There was no difference in the prevalence of non-ischaemic focal myocardial fibrosis between both groups (50.0% vs. 49.0%, *P* = 0.92). A greater burden of fibrosis trended towards the mild DCM group, but this did not reach statistical significance (3.5 ± 2.9 g vs. 7.4 ± 12.0 g, *P* = 0.09) (*Table 3*).

**Figure 3:**
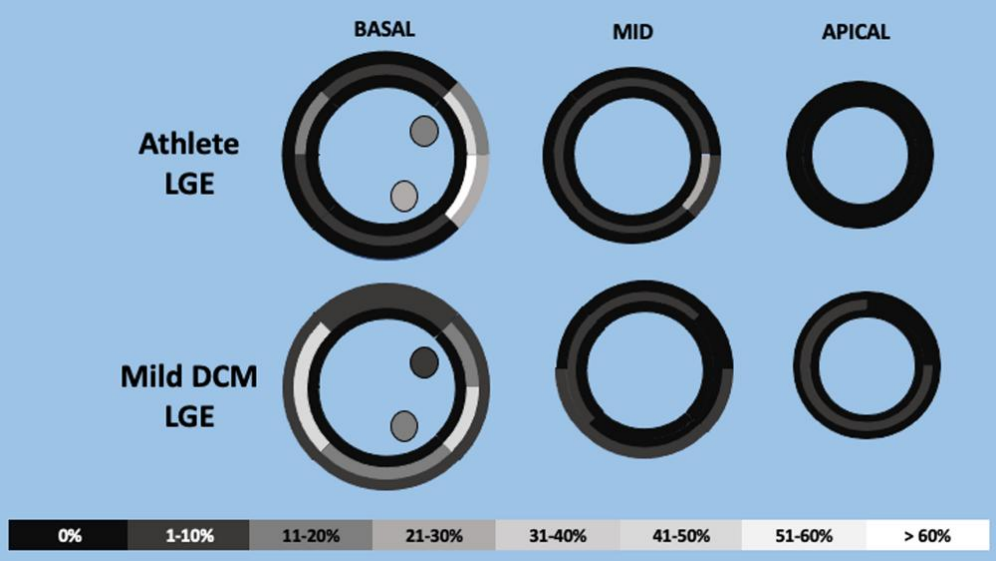
Fibrosis distribution in athletes compared with mild DCM patients. Athletes with fibrosis most commonly had mid-myocardial myocardial fibrosis affecting the basal inferolateral (87.5%) and anterolateral (53.1%) segments along with mid-inferolateral (25.0%) and subepicardial basal inferolateral segments (21.9%), but only 9.4% of athletes with fibrosis had fibrosis affecting the basal inferoseptal segment. Mild DCM patients with fibrosis most commonly had mid-myocardial myocardial fibrosis affecting the basal inferolateral (50.0%), inferoseptal (45.8%), and anteroseptal (45.8%) segments. DCM, dilated cardiomyopathy; LGE, late gadolinium enhancement.

The distribution of non-ischaemic fibrosis varied considerably between the groups (*Figure 3*). Athletes with fibrosis had a significantly greater prevalence of basal mid-myocardial inferolateral fibrosis than mild DCM patients (87.5% vs. 50.0%, *P* = 0.002), whereas basal mid-myocardial inferoseptal fibrosis was significantly more common in mild DCM patients than athletes (45.8% vs. 9.4%, *P* = 0.002). Furthermore, athletes had a greater prevalence of RVIP LGE (67.2% vs. 6.1%, *P* < 0.001), but there was no significant difference in LV papillary muscle fibrosis prevalence (31.3% vs. 22.4%, *P* = 0.30) between the groups.

**Figure 4:**
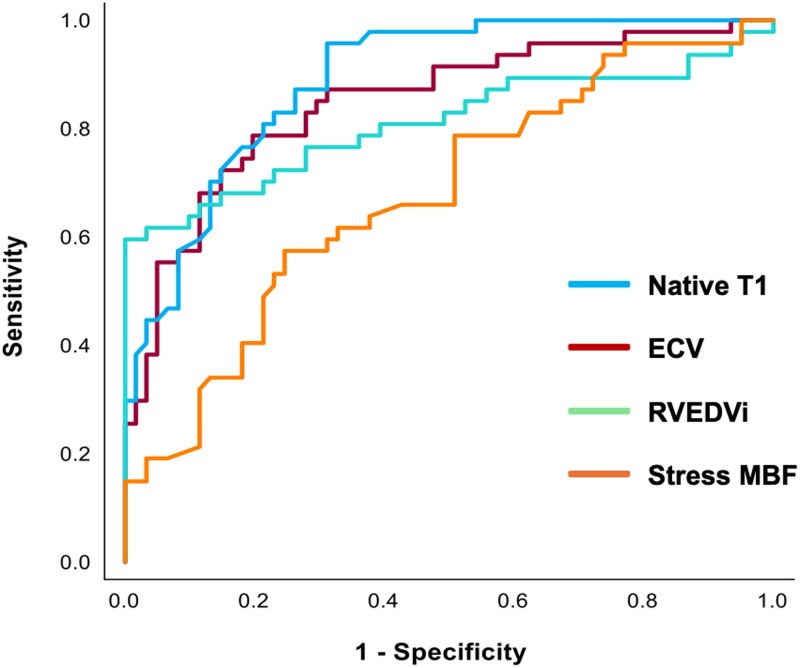
ROC analysis of native T1, ECV, RVEDVi, and stress MBF between athletes and mild DCM patients. ECV, extracellular volume; MBF, myocardial blood flow; ROC, receiver operating characteristic, RVEDVi; right ventricular end-diastolic volume indexed to body surface area.

On ROC analysis, native T1 [area under curve (AUC) 0.89, *P* < 0.001], ECV (AUC 0.85, *P* < 0.001), RVEDVi (AUC 0.81, *P* < 0.001), and stress MBF (AUC 0.68, *P* < 0.001) were all able to differentiate athletes and patients with mild DCM (*Figure 4*). The presence of non-ischaemic LGE was not discriminatory (AUC 0.50, *P* = 0.93).

## Discussion

In this prospective study, veteran endurance athletes with LV dilatation had a distinctive pattern of myocardial fibrosis along with greater RV dilatation and lower native T1 and ECV compared with patients with mild DCM. Athletes also demonstrated higher stress MBF and MPR than mild DCM patients.

### Fibrosis distribution

Despite no difference in the overall prevalence of myocardial fibrosis between athletes with ventricular dilatation and mild DCM patients, the distribution of myocardial fibrosis varied significantly. Athletes predominantly exhibited myocardial fibrosis affecting the basal lateral segments, whilst septal involvement was rare but common in mild DCM patients. Our findings therefore suggest that fibrosis involving the septum is a specific finding in patients with DCM and when found in an athlete with cavity dilatation may raise the suspicion of underlying cardiomyopathy. This is also in keeping with previous literature, which has demonstrated that myocardial fibrosis occurring in otherwise healthy athletes predominantly affects the basal lateral myocardial segments.^20,21^

Fibrosis involving the basal inferolateral segment was a common finding amongst both groups. The postulated mechanisms for the development of basal inferolateral myocardial fibrosis include LV pressure and/or volume overload causing an area of the myocardium, which is potentially more susceptible to high shear wall stress forces, to develop fibrosis.^12^ It is therefore plausible that the fibrogenic mechanisms in both athletes with physiological remodelling and those with DCM overlap.

Kübler *et al.*^22^ compared 40 top-level German athletes with 48 DCM patients. They found that the prevalence of myocardial fibrosis was significantly greater in DCM patients (44%) than athletes (5%) but did not compare the location of fibrosis. However, the groups were not matched for LV dilatation nor age, and DCM patients had significantly greater LV cavity volumes compared with athletes (132 ± 41 mL/m^2^ vs. 105 ± 17 mL/m^2^, *P* = 0.001) and were on average 29 years older than athletes. Furthermore, the study included patients with severe DCM (mean LVEF 29%) which may explain the significantly greater prevalence of fibrosis in DCM patients. Millar *et al.*^7^ compared 24 healthy athletes with LV dilatation and 34 patients with mild DCM. They found a much higher prevalence of myocardial fibrosis in mild DCM patients compared with athletes [17 (50%) vs. 0, *P* < 0.0001] but did not perform parametric tissue mapping nor report the distribution of fibrosis. The absence of fibrosis in athletes in this previous study may have been due to the inclusion of younger athletes (mean age 32.3 ± 10.4 years; range 18–58 years) compared with our study (mean age 58.8 ± 6.0 years), and the athletes they studied were predominantly runners (42%), which may have also affected fibrosis prevalence. This is pertinent as older male cyclists and triathletes are believed to be the group who exhibit the highest prevalence of myocardial fibrosis, and therefore this likely reflects the reason for the reported prevalence of fibrosis in our athlete cohort.^12^

### Native T1 and ECV

Athletes with cavity dilatation had significantly lower native T1 and ECV than mild DCM patients. Raised native T1 times and ECV in those with DCM have also been demonstrated in other studies suggesting evidence of diffuse fibrosis.^23–25^ These results are consistent with previous findings that athletic remodelling is associated with myocyte hypertrophy and thus lowered ECV as opposed to an increase in ECV, indicative of interstitial fibrosis, which occurs in cardiomyopathy.^26^

Native T1, ECV, and RVEDVi were superior to stress MBF as discriminators of athlete’s heart from mild DCM with high specificity and sensitivity. This is in keeping with a study by Mordi *et al*.^27^ who compared middle-aged males with early DCM and male athletes with mildly or borderline depressed LV systolic function using T1 and T2 mapping on CMR. They also found native T1 to be the best differentiator of these groups (AUC 0.91, *P* < 0.001). Therefore, native T1 may have a potential important diagnostic role for distinguishing athlete’s heart from DCM. However, in both our and the previous study by Mordi *et al.*, there was a considerable overlap of individual values between the groups, which may limit clinical utility.

### RV dilatation

Athletes had evidence of balanced ventricular dilatation involving both the RV and LV as opposed to mild DCM patients where lone LV ventricular dilatation was mainly present. These findings are in keeping with physiological athletic adaptation as the more compliant RV tends to preferentially dilate with chronic athletic training.^28^ RV dilatation is a well-established feature of athlete’s heart and is directly proportional to training intensity.^29^ In DCM, RV involvement may occur in ∼30% of DCM cases and is possibly related to the extent of LV systolic dysfunction.^30,31^ As our DCM cohort consisted of an early or mild form, this may explain why RV dilatation was not prevalent amongst those with mild DCM.

### Stress MBF and MPR

Stress MBF and MPR were lower in those with mild DCM than athletes with LV dilatation. A study by Gulati *et al.*^32^ displayed a similar reduction in stress MBF and MPR in DCM patients compared with controls, and these findings were believed to represent microvascular dysfunction. In their study, resting MBF was also raised in DCM patients, which was not replicated in our study. However, they included those with a more severe form of DCM as the mean LVEF of their DCM cohort was 35%. Therefore, increased resting MBF may be a feature of more advanced disease or decompensation where there is increased cardiac workload at rest. In contrast, exercise training in athletes has been shown to enhance peripheral microvascular function, and this may also extend to cardiovascular microvascular function.^33^

Stress MBF had the lowest AUC out of the parameters that were used to differentiate athletes with LV dilatation and mild DCM. Given that it requires specialized sequences and dedicated post-processing software along with the use of a stressor to induce hyperaemia, it may be argued that native T1, ECV, and RVEDVi are more time- and cost-efficient differentiators of athletic remodelling from mild DCM.

### Limitations

This study was limited by a relatively small number of participants within both groups of whom were imperfectly matched for baseline characteristics and LVEF. Our study also exclusively included veteran male participants, which limits clinical translation to other groups. However, older male athletes are believed to be the athletic group who are at most at risk of developing myocardial fibrosis and therefore justified our study selection.^12^

Neither group was genetically tested for cardiomyopathy, and it is not clear what proportion of seemingly healthy athletes actually expressed an underlying genetic DCM variant. Certain athletes with a mild DCM phenotype have been shown to possess cardiomyopathic genetic polymorphisms, and this has also been discovered in those with idiopathic fibrosis on post-mortem in those who have suffered sudden cardiac death.^34,35^ Therefore, an important area for future research involves combining advanced CMR and genetic testing in athletes who exhibit cardiac features consistent with a mild cardiomyopathy phenotype.

## Conclusion

Veteran male athletes with LV dilatation exhibited a pattern of myocardial fibrosis and CMR parametric tissue mapping characteristics, which were distinctive from patients with mild DCM. Whilst both groups exhibited a similar prevalence of non-ischaemic fibrosis, septal fibrosis was rare amongst athletes. Furthermore, RV dilatation was common in athletes but not seen in those with mild DCM. Native T1, ECV, and stress MBF were all able to differentiate between these cohorts, albeit with overlap between the groups.

Recognition of this pattern may be clinically useful to differentiate these two overlapping phenotypes. In particular, older athletes with septal fibrosis and LV dilatation without co-existing RV dilatation should prompt further investigation to exclude cardiomyopathy. Larger studies, particularly with longitudinal data and those combining advanced CMR techniques with genetic testing, are required to further investigate these findings.

## Data Availability

The data underlying this article will be shared on reasonable request to the corresponding author.
